# Epidemiology and risk factors of expatriates returning to Switzerland colonized at the intestinal level with multidrug-resistant Enterobacterales

**DOI:** 10.1007/s10096-025-05069-w

**Published:** 2025-02-14

**Authors:** Edgar I. Campos-Madueno, Claudia Aldeia, Marie C. Roumet, Andreas Limacher, Parham Sendi, Andrea Endimiani

**Affiliations:** 1 Institute for Infectious Diseases (IFIK), University of Bern, Friedbühlstrasse 25, Bern, CH-3001 Switzerland; 2 Department of Clinical Research, University of Bern, Bern, Switzerland; 3 Present Address: Functioning Information Reference Lab, Swiss Paraplegic Research, Nottwil, Switzerland

**Keywords:** *E. coli*, ESBL, Intestinal colonization, Africa, Asia, ST131

## Abstract

Living in high-endemic regions increases the risk of intestinal colonization by multidrug-resistant Enterobacterales (MDR-Ent). This study investigated Swiss expatriates residing abroad (≥ 3 months) to assess their colonization status upon returning to Switzerland. Selective culture-based methods were implemented to detect third-generation cephalosporins- (3GC-R), carbapenems- (CR), and colistin-resistant (COL-R) strains. Whole-genome sequencing was used to characterize antimicrobial resistance genes, sequence type (ST), and phylogroup of MDR-Ent. Epidemiological data were analyzed using uni- and multivariable models to identify risk factors, providing crude and adjusted odds ratios (ORs). Among 196 participants living across Africa, Asia, the Americas, and Europe, the overall MDR-Ent colonization prevalence was 42.9%. Continent of residence emerged as a significant risk factor (*p* = 0.04) for colonization: Africa (adjusted OR = 3.4, 95% CI 1.0–11.0) and Asia (adjusted OR = 4.7, 95% CI 1.5–15.0). Extended-spectrum β-lactamase-producing *Escherichia coli* (*Ec*) was the most frequent isolated species (*n* = 107 out of 119 Ent). Most 3GC-R-*Ec* possessed *bla*_CTX−M_ genes (*n* = 89; 83.2%) and pandemic lineages were frequent (e.g., ST69 and ST131, *n* = 18). No CR-Ent were detected, but some COL-R strains (*n* = 18; of which 15 *Ec*) harbored the *mcr-1.1* gene. Expatriates represent an understudied population at risk of MDR-Ent colonization. This population may contribute to the importation and potential dissemination of dangerous bacteria into low-prevalence countries, as shown in this Swiss study, warranting further investigation and surveillance.

**Supplementary Information:**

The online version contains supplementary material available at 10.1007/s10096-025-05069-w.

## Introduction

The rise of antibiotic-resistant bacteria poses an increasing threat to human health, with recent estimates attributing 1.3 million deaths to these pathogens annually [1]. Many healthy people (HP) may unknowingly carry such bacteria, facilitating their spread and thus exacerbating this crisis [2]. Of particular concern is gut colonization by multidrug-resistant Enterobacterales (MDR-Ent), including third-generation cephalosporins, carbapenems and/or colistin-resistant strains (3GC-R, CR, and COL-R, respectively). These pathogens are increasingly associated with heightened morbidity and mortality rates [2, 3]. Therefore, continuous surveillance is critical to uncover epidemiological trends, identify risk factors, and develop targeted strategies to mitigate their spread and health impact.

Globally, *Escherichia coli* (*Ec*) - particularly 3GC-R-*Ec* [including extended-spectrum β-lactamase (ESBL) producers] - is the leading pathogen associated with a significant burden of infections [4]. The prevalence of intestinal colonization with ESBL-*Ec* among HP in community varies greatly depending on geographic region [2]. For example, the average prevalence (i.e., ESBL-*Ec*) is estimated to be lower in European countries (6–7%) compared to regions in Africa (21.4%) and Southeast Asia (27%) [5, 6]. In contrast, intestinal colonization with CR-*Ec* in HP has been reported at prevalence of 0.1% in Switzerland, 2.9% in China, and 5.6% in India [7–9]. For COL-*Ec*, studies indicate highly variable spatiotemporal distributions among HP, with prevalence rates ranging from 2.4% to 11.5% in Taiwan and China to as high as 66.1% and 70.4% in Vietnam and Tanzania, respectively [10–13].

Travelers visiting highly endemic regions (e.g., Asia and Africa) are frequently colonized at the intestinal level with ESBL-positive and COL-R-*Ec*, whereas colonization with CR-Ent remains rare [2]. Notable examples include ESBL-Ent colonization rates of up to 80% among Swiss and Dutch travelers to Southeast Asia (e.g., India), with 42% of Dutch travelers specifically resulting colonized after visiting North African countries [14, 15]. Similarly, studies of Swiss travelers reported colonization with ESBL-*Ec* in 86.8% of people returning from India and 54% from Tanzania [16, 17]. Colonization with COL-R-*Ec* has also been observed following travel, contributing to the global spread of the *mcr* genes that confer resistance to polymyxins [18]. While less common than ESBL-*Ec* colonization, COL-R-*Ec* has been documented, for example, in travelers to Tanzania and in India (16.2% and 10.5% prevalence, respectively) [16, 19, 20].

Expatriates (or expats), represent another at-risk population due to their prolonged stays abroad. The epidemiology and risk factors for intestinal colonization with MDR-Ent in this population remain unexplored. Therefore, in this study, we examined a cohort of Swiss expats who had lived abroad for at least three months, assessing their intestinal colonization status upon returning to Switzerland. We employed culture-based selective methods and whole-genome sequencing to characterize the isolated MDR-Ent and investigated potential risk factors associated with their colonization.

## Materials and methods

### Participant enrollment and ethical approval

Swiss expats who were ≥ 18-year-old and living abroad (≥ 3 months) met the inclusion criteria for this prospective prevalence study (https://data.snf.ch/grants/grant/192514). Volunteers provided written informed consent and epidemiological questionnaires. An illustrative instruction for sampling was provided. Stool samples were self-collected in appropriate containers (Thermo Fisher Scientific) without preservatives (stored at 4 °C) within a maximum of 7 days of their return to Switzerland and sent immediately *via* regular mail to our institute (IFIK, University of Bern, Switzerland) for processing.

### Sample processing

Stool specimens (~ 50–100 µg) were screened for 3GC-R-, CR-, and/or COL-R-Ent by broth pre-enrichment culture-based methods as previously described [16, 21–23]. Specifically, stools were pre-enriched for 6 h at 36 ± 1 °C in Luria-Bertani (LB) broth containing cefuroxime (3 mg/L) or colistin (2 mg/L), followed by overnight plating on ChromID ESBL, CARBA SMART, or Colistin R chromogenic media (bioMérieux), respectively (referred henceforward as ESBL-, CARBA-, and COL-plates for simplicity). Bacterial colonies grown on the overnight plates were selected for species identification (ID).

### Species ID and antimicrobial susceptibility tests (ASTs)

ID was performed by matrix-assisted laser desorption/ionization time-of-flight mass spectrometry (MALDI-TOF MS). ASTs were performed by the broth microdilution method using the Sensititre GNX2F panels (Thermo Fisher Scientific) and interpreted according to the 2024 EUCAST criteria [24]. An intestinal colonization-positive screening result was defined when at least one 3GC-R-, CR-, and/or COL-R-Ent was isolated from selective plate(s). *Ec* strains with non-susceptibility to ≥ 1 agent in ≥ 3 antimicrobial categories were categorized as MDR [25]. Overall, based on the ASTs of each MDR-Ent and genome data (see below), only unique isolates were considered for downstream analyses.

### Whole-genome sequencing (WGS)

Strains were subjected to Illumina WGS on a NovaSeq 6000 instrument (2 × 150-bp paired-end), as previously described [23, 26, 27]. In short, raw reads were quality checked with FastQC v0.11.9 (https://github.com/s-andrews/FastQC) followed by adaptor trimming with Trimmomatic v0.36 (http://www.usadellab.org/cms/?page=trimmomatic) for paired-end data (ILLUMINACLIP: TruSeq3-PE-2.fa:2:30:10), both with default parameters. Construction of draft assemblies was done with Unicycler v0.4.8-v0.5.0 (https://github.com/rrwick/Unicycler) using preprocessed Illumina reads as input, followed by the removal of contigs shorter than 200-bp with SeqKit v2.5.0 (https://bioinf.shenwei.me/seqkit/). Quality of the resulting draft assemblies were assessed with QUAST v5.2.0 (https://github.com/ablab/quast) with default parameters.

### *In silico *strain characterization

Species ID was confirmed with the Type (Strain) Genome Server (TYGS; https://tygs.dsmz.de/) using the strains’ draft assemblies. Screening of antimicrobial resistance genes (ARGs), and sequence type (STs) were done with the tools of the Center for Genomic Epidemiology (https://genomicepidemiology.org/) command-line versions of ResFinderv4.1 (default parameters) and MLST v2.0. Phylogroup typing was determined with the online ClermonTyping tool (http://clermontyping.iame-research.center/) with default parameters. Identification of chromosomal polymyxin resistance mechanisms was performed by amino acid alignment with Geneious Prime v2023.0.4 using the translated gene sequences of *mgrB* (Gene IDs 946351 and 69611210), *pmrA* (Gene IDs 948631 and 69612466), *pmrB* (Gene IDs 948632 and 69612467), *phoP* (Gene IDs 945697 and 69610382), and *phoQ* (Gene IDs 946326 and 69610381) from *Ec* and *Enterobacter kobei*, respectively, available in NCBI (https://www.ncbi.nlm.nih.gov/gene/).

### Statistics and risk factor analyses

Results of the stool analysis were used to estimate the proportion of expats who were colonized with MDR-Ent with the associated 95% Wilson confidence interval (CI). Categorical variables were summarized as number and proportion, while continuous variables as median with quartiles. Group comparisons between people colonized and not colonized with MDR-Ent were performed using the non-parametric Mann-Whitney-Wilcoxon test for continuous variables and Chi-squared test for categorical variables.

Risk factors and predisposing conditions for colonization with MDR-Ent were assessed used uni- and multivariable mixed logistic regression, calculating crude and adjusted conditional odds ratios (ORs). To account for dependence of data within country, models included a random intercept for the country. In the multivariable model, we incorporated all characteristics for which the *p*-value associated to the crude (OR) was < 0.2 except “regular tap water consumption”. Notably, the characteristic “regular tap water consumption” (see Supplementary File 1) was excluded from the multivariable model since data presents counter intuitive results. Namely, drinking tap water was found to reduce the risk for colonization with MDR-Ent (crude OR = 0.4, Supplementary File 2). As such protective effect is highly unlikely, we believe that the information collected is not accurately reflecting the risk factor we intended to assess. The robustness of the selected multivariable model to small perturbations in the dataset (i.e., model stability) was assessed using 1000 bootstrap re-sampling with replacement, estimating the bootstrap inclusion frequency of each variable. All statistical analyses were conducted using R version 4.2.3 [28].

## Results

Between 2021 and 2023, a total of 196 participants with a median age of 48 years [interquartile range (IQR): 38–57] were enrolled in this study, of whom 46% were male (Supplementary File 1). All provided a stool specimen [i.e., *n* = 164 (83.7%) within ≤ 3 days], and all except one completed the epidemiological questionnaire.

### Work, travel and health characteristics

In total, 68% of the participants reported that they were employed by a Swiss embassy, while 22% were relatives or partners of Swiss embassy staff and 11% were other Swiss expats (Supplementary File 1). Participants reported living in a foreign city for a median of 1.7 years (IQR: 1.0, 2.6); recent travel (> 2 days in the last year) was reported by 73% of participants. The majority of people listed no hospitalizations during the last 2 years (90%), while 34%, 9.7%, 3.1%, and 51% stated antibiotic use during the last year, gastro-intestinal tract chronic disease(s), constant intake of stomach antacids or acid reducers, and diarrhea in the last 3 months, respectively. Of note, for all the aforementioned characteristics, there were no statistically significant associations (*p* > 0.05) with a positive intestinal colonization screening (Supplementary File 1).

### Prevalence of colonized expats

We identified an overall intestinal colonization prevalence of 42.9% [95% CI (36.1–49.9%)] (Table 1). Specifically, the colonization prevalence for HP living in Africa, Asia, the Americas and Europe were 48.5% [95% CI (36.9–60.3%)], 57.4% [95% CI (44.9–69.0%)], 29.4% [95% CI (13.3–53.1%)] and 21.6% [95% CI (12.5–34.6%)], respectively.

**Table 1:** Intestinal colonization prevalence in the overall study population and by continent in Swiss expats

Group	Total samples (*n*)	Positive samples (*n*) ^a^	Prevalence ^b^	95% CI ^c^
Africa	66	32	48.5%	[36.9–60.3%]
Asia	61	35	57.4%	[44.9–69.0%]
Americas	17	5	29.4%	[13.3–53.1%]
Europe	51	11	21.6%	[12.5–34.6%]
**Overall**	196 ^**d**^	84 ^**d**^	42.9%	[36.1–49.9%]

Note. n, sample size; CI, confidence interval

^**a**^ Number of stool samples that resulted colonization-positive (i.e., carrying MDR-Ent) after intestinal colonization screening

^**b**^ The proportion (percent) of study volunteers that are colonized at the intestinal level with MDR-Ent

^**c**^ The associated prevalence 95% Wilson confidence interval

^**d**^ The overall sample count included a participant (colonization-positive) that did not complete the epidemiological questionnaire (i.e., no country/continent was specified)

### Risk factors for colonization with MDR-Ent

A total of 27 potential risk factors (i.e., variables) were analyzed in uni- and multivariable models to determine crude and adjusted ORs, respectively (overall list in Supplementary File 2).

In the univariable models, a significant risk factor identified was the living continent (*p* = 0.003), with Africa [OR crude = 3.6, 95% CI (1.4–8.9)] and Asia [OR crude = 5.0%, 95% CI (2.0-12.3)], compared to Europe (baseline), as significant risks for gut colonization with MDR-Ent. Furthermore, the number of times per week having dinner outside the foreign home was also identified as a significant risk factor (*p* = 0.028). Other well-established risk factors (mentioned above), such as recent hospitalizations [OR crude = 0.5, 95% CI (0.1–1.5)], gastro-intestinal tract chronic disease(s) [OR crude = 0.4, 95% CI (0.1–1.3)], and diarrhea [OR crude = 1.2, 95% CI (0.6–2.3)] were not significantly associated with an increased risk for gut colonization with MDR-Ent.

Six potential risk factors were identified in the univariable analyses as having a *p* value < 0.2 and hence used for adjustment of the multivariable model. Bootstrap resampling indicated a rather good stability of this variable selection process. Namely, the inclusion frequency was found to be high for the selected variables (i.e. comprised between 62% and 95% for 5 of the 6 selected variables, 33% for the last one) and rather low for the non-selected variables (i.e. comprised between 0% and 41% for all except one variable, 65.3% for the last one) (Supplementary File 3). In the multivariable analysis, only the residing continent was found as having a significant effect for gut colonization with MDR-Ent (*p* = 0.04) (Supplementary File 2). In particular, Swiss expats living in countries within the African [OR adjusted = 3.4, 95% CI (1.0–11.0)] and Asian [OR adjusted = 4.7, 95% CI (1.5–15.0)] continents were associated with a high increase in risk of colonization with MDR-Ent.

### Features of the MDR-Ent colonizing the Swiss expats

**Fig. 1:**
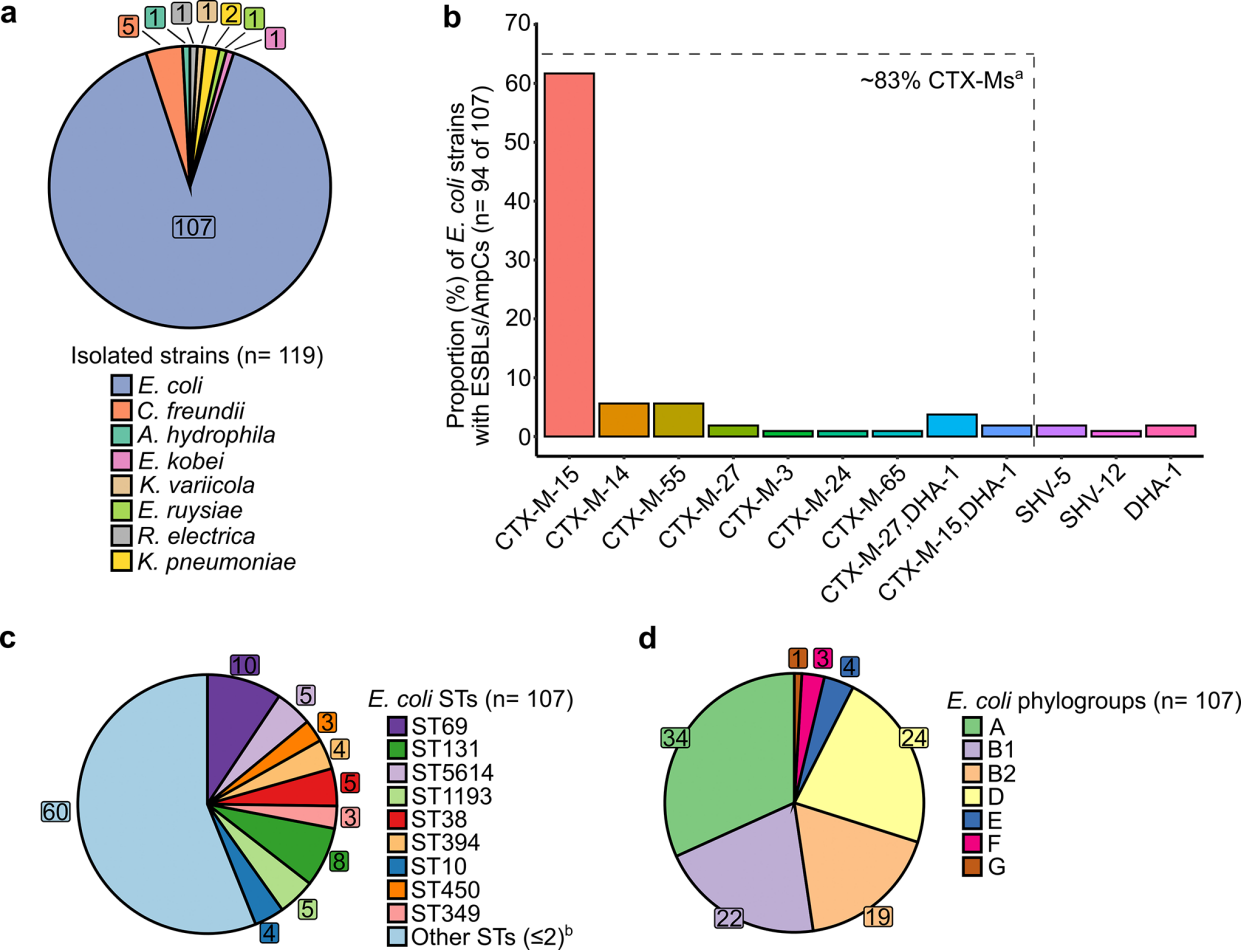
Genomic characteristics of the 119 strains isolated from 84 intestinal colonization-positive samples in this study. In **a**) we show the number of isolated strains confirmed at the species-level by whole-genome sequencing. In **b**) the proportion of *E. coli* strains associated with ESBL and/or AmpCs encoding genes (*n* = 107) are shown. In **c**) we display the number of *E. coli* strains per sequence type (STs), while in **d**) the distribution of phylogroups for all *E. coli* samples (*n* = 107). Source data are provided as a *Source Data* file. ^a^ Percentage of *E. coli* strains possessing ESBL and/or AmpCs encoding genes (*n* = 94) associated with a CTX-M encoding gene. ^b^ Total number of *E. coli* strains where STs were shared between 2 strains or were unique (*n* = 1)

A mean of 1.4 MDR-Ent were isolated from 83 Swiss expats (119 MDR-Ent in total) (Supplementary File 4). The main MDR-Ent isolated was *Ec* (107 out of 119 strains) (Fig. 1a). Notably, a total of 57 of 107 strains were MDR-*Ec*, showing resistance primarily to 3GCs (*n* = 52, 48.6%), trimethoprim-sulfamethoxazole (*n* = 41, 38.3%) and ciprofloxacin (*n* = 33, 30.8%) (Supplementary File 4). No CR-*Ec* were observed, whereas 15 of 107 *Ec* displayed a COL-R phenotype (14.0%). Seven of the 15 COL-R-*Ec* were also MDR (two 3GC-R), while 8 were non-MDR.

The main mechanism of resistance against β-lactams presented by *Ec* strains was the production of CTX-M-type ESBLs (89 out of 107 *Ec*; 83.2%), consisting primarily of CTX-M-15 (*n* = 66; 61.7%) (Fig. 1b, Supplementary File 4). Similarly, non-*Ec* strains (*n* = 12) such as *Citrobacter freundii* and *Klebsiella* spp. carried CTX-M-, CMY- and SHV-type β-lactamases. A cryptic *Escherichia ruysiae* clade IV strain (S1-IND-07-A) possessing CTX-M-15 was also identified (Supplementary File 4) [27]. As for COL-R mechanisms, *mcr-1.1* was found in 8 of 15 COL-R-*Ec* (53.3%). Likewise, mutations in the chromosomal regulators *mgrB*, *pmrA/B*, and *phoP/Q* were identified in COL-R-*Ec* that carried no *mcr* genes (Supplementary File 4). Three other non-*Ec* were also COL-R, an *mcr-3*-like associated *Aeromonas hydrophila* strain, and 2 other *Ent* [e.g., *Raoultella electrica* (S2-IND-01-C)] lacking MCRs, but carrying chromosomal mutations [26].

### Clonality of *Ec* strains

A total of 56 unique *Ec* STs were observed, with ST69 (*n* = 10) and ST131 (*n* = 8) being the most common ones identified (Fig. 1c; Supplementary File 4). In particular, strains of ST69 were predominantly CTX-M-type producers (*n* = 9), with CTX-M-15 being the most common variant (*n* = 6) isolated from Swiss expatriates living in European (*n* = 4), African (*n* = 3) and Asian (*n* = 3) countries. In contrast, ST131 *Ec* consisted mainly of CTX-M producers (*n* = 6), including a CTX-M and DHA-1 co-producer, and an MDR COL-R-*Ec* isolated from Swiss expatriates living in African (*n* = 3) and Asian (*n* = 4) countries, but also from a volunteer living in Venezuela (Supplementary File 4). Overall, the most often isolated phylogroups among all 107 *Ec* were A (*n* = 34; of which 4 ST10), D (*n* = 24; of which 10 ST69) and B2 (*n* = 19; of which 8 ST131 and 5 ST1193) (Fig. 1cd; Supplementary File 4).

## Discussion

The prevalence of intestinal colonization by MDR-*Ec*, particularly those producing ESBLs or pAmpCs of the CTX-M- and/or DHA-type, respectively, is a growing concern [2]. Individuals exposed to high-endemic regions may face an increased risk, with expatriates representing an understudied at-risk group.

In this study, we identified a low prevalence of MDR-Ent, particularly ESBL-*Ec*, among 196 Swiss expatriates living in European countries (21.6%) (Table 1). Although this is higher than earlier reports in community people (e.g., 6% in Europe [5]), it remains significantly lower than the markedly higher prevalence observed among Swiss expatriates based in African and Asian countries, 48.5% and 57.4%, respectively [2, 5]. This contrast highlights the considerable regional differences in intestinal colonization prevalence, supporting that exposure to high-endemic regions may be a significant risk factor (see below) [2]. In this context, the higher prevalence of MDR-Ent possessed by Swiss expats residing in European countries may reflect the possibility that such expats are not representative of the standard HP community in European countries, as this special population (i.e., Swiss expats mostly working for diplomatic institutions) may be more often involved in travel activities [e.g., 73% traveled more than > 2 days within the last year (Supplementary File 1)].

In our analysis of 27 potential risk factors, we aimed to better understand the epidemiological mechanisms driving intestinal colonization with MDR-Ent in Swiss expatriates. Using uni- and multivariable mixed models, we found that only expatriates residing in African and Asian countries had a significantly increased risk of colonization (ORs adjusted, *p* < 0.05, 3.4 and 4.7, respectively) (Supplementary File 2). This finding aligns with numerous studies identifying endemic regions as key hotspots for acquiring MDR organisms (e.g [8, 10, 12, 14, 16, 21, 22]). Interestingly, other well-established risk factors, such as diarrhea, antibiotic use, or hospitalization(s), were not significant (both crude and adjusted ORs) [2, 29, 30]. While these results may be influenced by the limited sample size in our study, they provide valuable insight, but warrant further investigation.

Among Swiss expatriates colonized with MDR-Ent, *Ec* was the most frequently isolated species (89.9%), with a majority being ESBL producers of the CTX-M-type (Fig. 1a; Supplementary File 4). Notably, approximately 53% of *Ec* strains were MDR, displaying not only resistance to 3GC-Rs, but also co-resistance to other antimicrobial classes such as trimethoprim-sulfamethoxazole and ciprofloxacin, which are crucial for treating urinary tract and other common bacterial infections in the community [3].

The identification of *bla*_CTX−M_ genes as the main mechanism responsible for 3GC-R, particularly the *bla*_CTX−M−15_, is consistent with other studies, which have emphasized their global widespread (i.e., typically associated with plasmids), and high-level resistance to 3GCs [31]. Notably, in our study, *bla*_CTX−M−15_ was highly prevalent in *Ec* isolates (61.7%) from African and Asian countries, further corroborating findings from previous surveys (Fig. 1b; Supplementary File 4) (e.g [16, 17, 21, 22]). In such isolates, for example, the *bla*_CTX−M−15_ along with other ARGs, was commonly associated to well-know and conjugative IncFIA/B, IncFII, and IncB/O/K/Z plasmids [32].

Interestingly, we previously identified a rare *E. ruysiae (*cryptic clade IV*)* possessing an IncB/O/K/Z plasmid carrying *bla*_CTX−M−15_, in a Swiss expatriate living in India [27], supporting our observation that certain endemic regions may be risk factors for the colonization of rare and potential pathogenic bacteria (see below). Consistent with previous surveys, where intestinal colonization with CR-Ent colonizing HP (e.g., travelers) is rare [2], no carbapenemase producers were detected in this study. Additionally, we observed a smaller, but important, proportion of COL-R-Ent, mostly consisting of *Ec*, alongside rarer species like *R. electrica*, also isolated from a Swiss expatriate living in India (Supplementary File 4) [26]. As noted in previous surveys, particularly those involving travelers to Asian countries (e.g [19–21]), *Ec* isolates were associated either with the *mcr-1.1* or carried amino acid substitutions in key chromosomal regulatory encoding genes (e.g., MgrB V8A) associated with polymyxin resistance (Supplementary File 4) [33, 34].

Overall, the *Ec* strains colonizing the intestinal tracts of Swiss expatriates exhibited distinct epidemiological patterns, with many *Ec* belonging to well-known pandemic clones (e.g., ST69, ST131); these clones are frequently implicated in outbreaks and are known for causing difficult-to-treat infections (Fig. 1c; Supplementary File 4) [35–38]. Although no specific associations were observed between the continent of residence and the ST-types, the widespread detection of top pandemic clones across expatriates in Africa, Asia, and Europe underscores their global dissemination. Moreover, these hyperepidemic *Ec* clones often harbored *bla*_CTX−Ms_ and/or *bla*_DHAs_ and exhibited MDR phenotypes, further emphasizing their clinical importance. On this context, the B2 phylogroup, which included extraintestinal pathogenic *Ec* clones such as the CTX-M-producing ST131, was primarily isolated from expatriates based in Asia and Africa (Fig. 1d; Supplementary File 4). ST131 presents a considerable public health concern [36]. Its presence in the intestinal tracts of expatriates warrants attention, as this population may unknowingly facilitate the importation and continuous expansion of this lineage in their home or host countries.

In conclusion, our findings suggest that HP living long-term (≥ 3 months) in countries on the African and Asian continents may face an elevated risk of intestinal colonization by dangerous MDR-Ent, particularly CTX-M-producing *Ec* belonging to pandemic clones. The high overall colonization rates observed among Swiss expatriates may reflect characteristics of the specific population studied and the sample size. Nonetheless, this concerning trend, coupled with the potential impact of MDR-Ent importation into low-prevalence countries, underscores the need for further investigation to better understand and mitigate these risks. Finally, we emphasize the critical importance of intestinal colonization screening in both expatriates and travelers, as these populations may introduce clinically important MDR-Ent and other relevant bacteria (e.g., Gram-positive species) in low-prevalence countries. Such an approach could help identifying the early importation of high-risk bacterial clones.

## Electronic supplementary material

Below is the link to the electronic supplementary material.

Supplementary Material 1

Supplementary Material 2

Supplementary Material 3

Supplementary Material 4

Supplementary Material 5

## Data Availability

Data is provided within the manuscript.
